# Deriving proxy life cycle assessment datasets for manufacturing machines through data clustering

**DOI:** 10.1007/s00170-026-18236-w

**Published:** 2026-05-06

**Authors:** Nathan Dodd, Lenny Koh, Rachael Rothman

**Affiliations:** 1 The University of Sheffield School of Chemical, Materials & Biological Engineering, Mappin Street, Sheffield, S1 3JD UK; 2 The University of Sheffield Management School, Conduit Road, Sheffield, S10 1FL UK

**Keywords:** Life cycle assessment, Machines, Tools, Simplified methodology, Categorisation

## Abstract

Conducting Life Cycle Assessments (LCAs) for machines and tools in manufacturing is often time-intensive and hampered by difficulties in accessing suitable datasets. It is, nevertheless, important to consider the life cycle impacts of the machines and tools in a manufacturing process to identify hot spots, evaluate the relative importance of their impacts and develop strategies for further environmental improvements. This core novelty of this study is in the investigation of whether heterogeneous life cycle assessment (LCA) inventory and impact data for manufacturing machines can be meaningfully clustered, and whether the resulting groupings can be interpreted and operationalised as generalised proxy life cycle inventory datasets. It further evaluates the practical usefulness of these cluster-derived proxy datasets for supporting screening-level LCA and early-stage sustainability decision-making in manufacturing contexts. Three proxy LCA categories were developed, capturing broad similarities between machines while retaining sufficient detail for high-level sustainability assessments. The resulting machine proxy data provides a practical tool for streamlining LCA decision-making, allowing practitioners to estimate life cycle impacts from production to end-of-life even in the absence of detailed datasets. Validation against individual ecoinvent datasets showed that these generalised categories produce reasonably accurate approximations within typical uncertainty ranges, supporting exploratory analyses and screening applications. However, they are not intended to replace full LCAs for specific machines where precise assessment is required. Future work could enhance the proxy datasets by incorporating real-time operational data and regional variations, potentially using machine learning to refine impact estimates dynamically. Industrial integration of these datasets, such as in digital twin models or automated LCA platforms, would enable rapid, scalable sustainability assessments, supporting more informed decision-making in machine selection, procurement, and operational planning.

## Introduction

Life Cycle Assessment (LCA) is the most widely used method for measuring the environmental impacts of a product or process. It evaluates the entire life cycle of a product, from raw material extraction through the use phase and ultimately to the product’s end-of-life and disposal. LCA is standardised by ISO 14040 [1] and ISO 14044 [2]. However, the consistency and reliability of results are highly dependent on the quality of the available data and specificity of the analysis being conducted. In manufacturing contexts, striking the right balance between detail and simplicity is critical: too much complexity slows down decision-making, while too little risks overlooking key impacts. By streamlining LCA steps where appropriate, manufacturers can save substantial time in evaluating alternative processes and materials, enabling faster design iterations and more agile sustainability assessments.

LCA utilises distinct data types to model environmental impacts across a product or system’s life cycle. Foreground data refers to the primary data which defines the system being studied. The European Commission [3] defines primary data as “directly measured or collected data representative of activities at a specific facility or set of facilities.” In the context of LCA, foreground data includes raw inputs from manufacturing processes, data obtained from suppliers and distributors, and usage-phase data reflecting how the product is used by consumers. This data type is critical for accurately representing the systems directly influenced by the study and forms the foundation for tailored, high-resolution environmental assessments. In a manufacturing context, foreground data capture the material, and energy flows specific to individual machines, production lines, and factory operations, ensuring that assessments reflect the actual performance of industrial processes.

Background data represent secondary information for processes outside the immediate control of the study, such as energy generation or raw material production. These are typically sourced from established life cycle inventory (LCI) databases to ensure system completeness and consistency. Proxy data are substitute datasets used when specific data are unavailable, selected based on technical, functional, or compositional similarity to the missing process or material [4]. Proxy data if often used when there is no readily available data for a specific machine or tool that is a component of the process being analysed. Proxy data are especially important in prospective or early-stage LCAs, where empirical data for emerging technologies or unique systems may not yet exist. Their use enables continuity in modelling and supports comparative assessments or preliminary environmental evaluations and ensures components are not completely excluded from assessment. While the introduction of proxy data can increase uncertainty, transparent documentation and careful justification of their selection enhance the robustness and credibility of the analysis. As more accurate data become available, proxy data can be refined or replaced, enabling iterative improvement of the assessment [5].

Gathering detailed data for industrial machinery is difficult, making full LCAs resource-intensive and time-consuming [6]. Databases, such as Ecoinvent [7] can be used to obtain secondary data for processes and materials, however, secondary datasets often lack the required specificity and quality, making suitable proxy data difficult to identify. Research continues to be undertaken with the aim of achieving better integration of data into the LCA method with Julian Baehr [8] presenting the requirements of future reporting to be built on large, high quality and robust datasets.

These limitations pose significant challenges to conducting timely and reliable environmental assessments of industrial processes, particularly where diverse and complex machinery, tools, and manufacturing methods are involved in producing the final product. This study addresses these challenges by providing alternative datasets derived from a broad range of currently available machine data. Through a data-driven analytical approach that emphasises numerical characteristics over nominal classifications, Three generalised machine data categories are developed. These categories are designed to streamline Life Cycle Assessment (LCA) processes by significantly reducing the time and resources typically required for data collection and analysis. In practice, this allows manufacturers to evaluate environmental performance more quickly across different machine configurations, without needing bespoke datasets for each case. The approach therefore supports faster trade-off analyses during process planning and technology selection.

The study addresses the following research questions, considering both methodological novelty and real-world applicability:

- To what extent can life cycle assessment (LCA) inventory and impact data for manufacturing machines be meaningfully clustered, given the inherent heterogeneity and complexity of such datasets?
- Can the resulting clusters be interpreted and operationalised as generalised proxy life cycle inventory datasets for manufacturing and engineering machines?
- How effective and practically useful are these cluster-derived proxy datasets for supporting screening-level LCA and early-stage sustainability decision-making in manufacturing contexts?

## Literature review

Data requirements in LCA vary by methodological approach, which can include process-based LCA, input–output analysis (IOA), and hybrid models. Each approach has flexibility to be used across a range of industrial contexts including manufacturing [9]. As noted by Guinée et al. [10], the field is characterised by diverse and often competing terminologies and methodologies. Valente et al. [11] highlights these inconsistencies as barriers to broader LCA adoption and emphasise the need for improved data accessibility and interoperability.

The suitability of each method depends upon the requirements of the research question. Process-based LCA enables detailed comparisons between products or production methods, while input–output analysis (IOA) offers broader, system-level insights but is less suited for comparing similar products within a sector [12]. Advancements in digitalisation and data sharing are expected to enhance LCA methods, supporting more accurate and real-time evaluations [13, 14].

The scenario-based LCA method described by Ebrahimi & Koh [15] is used to compare specific materials and processes within a system. Modelling material scenarios helps balance cost, emissions, and compliance risks. This approach therefore has a high reliance on using accurate data for the materials and process options which are being investigated.

Input–output analysis (IOA) links environmental emissions to economic activities, making it useful for estimating the environmental footprint of machinery. This system-wide approach captures both upstream and downstream supply chain impacts [16]. However, the method is reliant on national and regional economic datasets, and the input–output analysis method often lacks granularity, making it unsuited for analysing specific machine components [17]. There is also a tendency that results may vary significantly based on the geographic context [18].

Streamlined LCA methods aim to reduce the complexity of a full process LCA by focusing on key impact categories or life cycle stages [19]. A streamlined LCA is heavily reliant on secondary data and predefined tools within LCA software to produce rapid results. This streamlined approach is more useful for initial hot-spotting and testing conclusions and for quick decision-making during the design or purchasing phase. Streamlined LCAs focus on key materials or stages with the highest impacts [20] and uses default datasets and assumptions to fill data gaps [21].

Performing Life Cycle Assessment (LCA) remains challenging due to data quality gaps, methodological complexities, and time-intensive processes. Karuppiah, Sankaranarayanan, & Ali [22] found that problems with the quality of data was the most critical challenge for LCA practitioners. Lueddeckens et al. [14] highlights the difficulty of ensuring reliable, comprehensive data, especially when relying on secondary sources, while Guinée et al. [10] and Ottinger et al. [23] emphasise how inconsistent methodologies and poor data management further complicate assessments. Dunn et al. [24] propose improved data structures, yet traditional LCA methods still demand significant time and resources.

In a study by Balcioglu et al. [25], the age of GWP data of materials was typically from the year 2010 however some datasets were as old as 1998. Data availability, quality, and scalability represent core challenges within the broader issue of data limitations in LCA and are considered to be especially important when modelling the foreground system [26]. When conducting prospective life cycle assessments (LCAs) of emerging technologies, three principal challenges are commonly identified: ensuring comparability across systems, addressing data limitations, and managing uncertainty [27]. A study by Moutik [28] further identified these concerns outlining issues around dataset completeness, timeliness of data sources, and overall data reliability, whilst Steffen Foldager Jensen summarised an overall low maturity of data for product life cycle decision-making [29]. These challenges point to the need for simplified, efficient tools to make LCA more accessible and practical for industrial use.

A critical challenge in LCA of manufacturing systems is the selection of appropriate background data, particularly given constraints on time, data quality, and modelling effort. Manufacturing equipment spans a continuum from general-purpose to highly specialised machinery; however, LCA databases, such as Ecoinvent, offer only a limited selection of machine-related datasets. Practitioners must navigate the trade-off between the convenience of generic modules, often lacking specificity, and the resource intensity of developing primary machine-level inventories. High-resolution data is essential when the machine constitutes the primary focus of the assessment, whereas lower-resolution proxies may suffice when machinery represents only a subsystem within a broader product system. Manufacturing LCAs have demonstrated the value of applied, machine-level data for identifying hotspots and supporting practical improvements [30]. However currently there is a shortfall of standardised, generalised and reliable machine datasets to support early-stage or screening-level LCAs.

Secondary data found in databases often has limitations, including being misleadingly named, over simplified or overly homogenised. The following are examples from the ecoinvent [7] database, however, it should be noted that these issues are prevalent across all secondary database sources.

Datasets can have a discrepancy between the data which is implied by the title of the entry and the real source of the data. The data for an *‘Industrial machine, heavy, unspecified {RER}| production’*, is based on a ‘Nordberg HP400 SX’ rock crusher which was produced in the USA in 1999. this dataset requires the assumption that any heavy industrial machine is, per kilogram, the equivalent of a rock crusher from 1999. A note within the description notifies the user of this datasets tenuous reliability, reading that ‘in cases where this component will have a high importance it would be unsuitable for use.’

There are numerous examples of oversimplified datasets, for example, the data listed for the component ‘*Building machine {RER}| production’* includes a description note that this component is based on a fictitious machine made from 100% of steel and that the energy used to produce the machine is equivalent to the energy used in the production of a car. Another example of an oversimplified dataset is the data for ‘*Helicopter {GLO}| production*’ in which a 1 tonne helicopter is listed as being manufactured from 500 kg of steel, 500 kg of aluminium and no amount of energy used for processing. These examples demonstrate a clear distinction between the complex reality of manufactured machines, with an array of components, processing and supply chain variables, and the many overly simplified datasets which are currently available.

Lastly there is a limitation relating to homogeneity, in which an array of distinct machines and tools are considered as a group rather than individually. In the case of ‘*printed wiring board mounting facility construction, surface mounting line GLO’* the data represents a set of machines used in a single production line. These are listed as including three smaller component placing machines, two larger components placing machines, one inspection device and one reflow oven. These modules are intended to be used where their overall contribution and importance to the analysis is relatively insignificant. The modules provide quick and easy solutions to users who desire to add more details for their overall system without performing time intensive studies. These modules demonstrate the role of more generic datasets, whilst highlighting the issues and limitations which currently exist within available datasets.

This study evaluates pre-existing datasets within the ecoinvent database to understand trends within machines and tools, then classifies those trends to develop a novel category methodology. The methodology is verified by comparing category outputs with detailed machine datasets.

### Uncertainty within LCA datasets

All data used within a life cycle assessment (LCA) inherently carries assumptions and associated uncertainty. As a result, any single numerical output from an LCA should be interpreted as an estimate rather than a precise or definitive value. Current literature suggests that for relatively well-characterised and specific datasets, uncertainty levels may be on the order of approximately 10%. However, as datasets become increasingly generic, particularly when they are used to represent broad classes of products or processes, the associated uncertainty can increase substantially, in some cases reaching 40% or more [31].

**Fig. 1:**
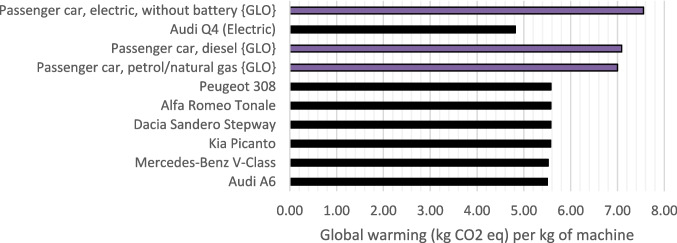
Comparison of reported NCAP production carbon emissions and ecoinvent datasets (ecoinvent = purple, NCAP = black)

A clear illustration of this effect can be observed when comparing reported carbon emissions from vehicle production based on manufacturer-specific or regulatory datasets (e.g. NCAP-related disclosures [32]) with the generalised vehicle production datasets available in the ecoinvent database. These comparisons reveal systematic differences in results. In particular, ecoinvent datasets tend to overestimate carbon emissions on a per-kilogram-of-vehicle basis. For example, the *Passenger car, petrol/natural gas* dataset reports values that are, on average, approximately 20% higher than those reported by NCAP. More pronounced discrepancies are observed for electric vehicles, where the difference between the *Passenger car, electric, without battery* dataset and the reported production emissions for a specific model such as the Audi Q4 can approach 40% (Fig. 1).

These discrepancies highlight the fundamental distinction between generalised background datasets and highly specific, product-level inventories. In this context, the present study seeks to develop proxy datasets representing broad categories of machines rather than individual, fully specified products. The resulting datasets are intended to provide illustrative; order-of-magnitude estimates of the sustainability profiles that an unknown or partially specified machine might reasonably be expected to exhibit. They are not intended to replace, nor to serve as a shortcut for, a comprehensive LCA of a specific machine or product, but rather to support comparative analysis and early-stage decision-making where detailed data are unavailable.

## Methods

### Clustering of LCA data for manufacturing machines

Ecoinvent is a comprehensive life cycle database that compiles datasets from a wide range of industrial, academic, and commercial sources. It contains approximately 22,000 individual datasets, covering a broad spectrum of materials, energy systems, transport processes, and manufacturing activities. The data used in this study is from the ecoinvent database and is for the cut-off system model. Comprehensive searches were conducted manually within the database with the aim of finding machine and tool datasets that perform some kind of mechanical function. Each dataset is based on embodied emissions, which is the impacts of producing the machine rather than the impacts related to the machines usage.

Data categories, including transport, appliances, construction infrastructure, agricultural infrastructure and energy infrastructure were searched to identify appropriate machine datasets, as well as by using key terms such as; machine, tool and engineering. The search was carried out manually as the underlying datasets are not consistently labelled in a manner that would enable the reliable identification of records representing machines or mechanically functional systems; such classifications are only implicitly conveyed through the dataset titles rather than through structured metadata.

A total of 43 LCA datasets were found which represent a range of different technologies and purposes. 40 of these datasets have been used in this study, as listed in Table 1. The other three datasets were identified as statistical outliers using the Local Outlier Factor (LOF) algorithm (n_neighbors = 5, contamination = 0.05). LOF evaluates the local density of each observation relative to its nearest neighbours, flagging observations that exhibit substantially lower density as potential outliers. Based on this criterion, the following datasets were identified: Coffee maker {GLO}, Computer, laptop {GLO}, and Printer, laser, colour {GLO}. These datasets display impact profiles that differ markedly from the majority of machines in the sample, indicating atypical environmental characteristic patterns within the feature space used for clustering.

**Table 1:** List of machines and tools used in analysis from ecoinvent (40 machines)

Ecoinvent name of dataset in form: machine name {Geographical Basis} *{RoW} is Rest of World, {CH} is China, {GLO} is Global, {RER} is Europe excluding Russia*			
Agricultural machinery, tillage {RoW}	Electric bicycle {GLO}	Hydraulic digger {RER}	Passenger car, diesel {GLO}
Agricultural machinery, unspecified {CH}	Electric kettle {GLO}	Hydraulic digger {RoW}	Passenger car, electric, without battery {GLO}
Agricultural machinery, unspecified {RoW}	Electric motor, vehicle {RER}	Industrial machine, heavy, unspecified {RER}	Printed wiring board mounting facility, surface mounting line {GLO}
Agricultural trailer {GLO}	Electric motor, vehicle {RoW}	Industrial machine, heavy, unspecified {RoW}	Printer, laser, black/white {GLO}
Bicycle {GLO}	Electric scooter, without battery {GLO}	Internal combustion engine, passenger car {GLO}	Refrigeration machine, R134a as refrigerant {GLO}
Building machine {RER}	Electronic component machinery, unspecified {GLO}	Internet access equipment {GLO}	Refrigerator {GLO}
Computer, desktop, without screen {GLO}	Hair dryer {GLO}	Keyboard {GLO}	Television {GLO}
Cookstove {GLO}	Harvester {CH}	Liquid manure tank trailer {GLO}	Tractor, 4-wheel, agricultural {GLO}
Dishwasher {GLO}	Harvester {RoW}	Locomotive {RoW}	Vacuum cleaner {GLO}
Disk drive, CD/DVD, ROM, for desktop computer {GLO}	Helicopter {GLO}	Metal working machine, unspecified {RER}	Washing machine {GLO}

Each of the remaining datasets fits within the definition of being a manufactured item which would be used as a machine or tool involved in the function of turning energy into useful work. Because the method draws on a wide range of machine and tool datasets, it directly reflects the diversity of equipment found in manufacturing environments. This breadth ensures the results are grounded in real production contexts, making the approach well aligned with applied manufacturing analysis. The environmental impact data per kg of each module was calculated using the ReCiPe midpoint (H) methodology [33].

Geographical scope in LCA datasets reflects region specific factors such as; energy mix, material supply chains, and manufacturing practices, which can significantly influence environmental impact results. As a result, datasets with different geographical bases (e.g. {CH}, {RER}, {GLO}, {RoW}) may not be directly comparable, and variations in impacts may arise from regional conditions rather than differences in the machines themselves.

### Ward’s hierarchical clustering method

Ward’s hierarchical clustering method is a widely used agglomerative clustering technique, first introduced by Ward in 1963 [34]. Unlike distance-based linkage methods such as single or complete linkage, Ward’s method explicitly aims to minimise the total within-cluster variance at each step of the agglomeration process [35]. By merging the pair of clusters that results in the smallest increase in the error sum of squares, the method produces clusters that tend to be compact, spherical, and relatively balanced in size [36]. This variance-minimising property has made Ward’s method particularly popular in applications where the preservation of cluster homogeneity is a key objective.

Ward’s method is based on a variance-decomposition framework in which the total inertia of the dataset is partitioned into within-cluster and between-cluster components. At each agglomeration step, clusters are merged such that the resulting increase in within-cluster variance is minimised. This least-squares optimisation principle provides a clear interpretation of the algorithm’s objective and justifies the use of Euclidean distance metrics. In this study, hierarchical clustering is implemented using the scipy.cluster.hierarchy library in Python.

A k-means clustering trial was performed to compare results with Ward’s method. Identical cluster allocations were obtained for k = 2, 3, and 4, indicating strong structural consistency between the two algorithms at these levels of partitioning. However, at k = 5 the solutions diverged, suggesting that the additional subdivision introduces instability or method-dependent differences in how the data are partitioned.

Before clustering, the raw ReCiPe results were standardised using *StandardScaler* to remove the influence of different units and numerical scales across the 18 impact categories. For each indicator, the average value across all machines was calculated and then subtracted from every machine’s result. The values were then divided by the standard deviation, which rescales them according to how much variation exists in that indicator.

After this transformation, each impact category has an average value of zero and a comparable spread. The numbers no longer represent absolute environmental impacts, but instead indicate whether a machine performs above or below the average for each category, and by how much. This ensures that all impact categories contribute equally to the clustering, rather than categories with larger raw magnitudes dominating the results.

### Derivation of proxy datasets from clustered LCA data

The objective of the clustering analysis was to identify three or four clearly distinct machine groups. A key purpose of developing proxy datasets is to ensure that they remain straightforward to interpret and practical to apply, while still providing meaningful estimation accuracy. Increasing the number of clusters would require more narrowly defined classification criteria, which are not sufficiently supported by the available dataset.

Three categories were ultimately selected based on the evaluation of clustering quality metrics and the need to balance analytical robustness with usability. As the number of categories increases, the classification becomes more complex and less suitable for screening-level assessments. At the same time, dividing the dataset into additional clusters reduces the number of observations within each group, which can weaken the statistical stability of the derived proxy profiles.

The selection of three categories therefore reflects a compromise: a small number of distinct and interpretable groupings that broadly capture differences in machine complexity. These range from relatively simple machines, characterised by bulk materials and limited component diversity, to progressively more complex machines exhibiting greater material and functional heterogeneity.

An average environmental impact profile is calculated for each cluster (as later defined in Sect. 4.2) by aggregating the environmental impact metrics of all machines ${X}_{m}$ assigned to that cluster $c$. The final environmental impact profile for each machine category, $\overline{{X }_{c}}$, is calculated by Eq. 1. Simply put, the mean result is taken across each impact category for each cluster.

Equation 1 Category-level aggregation of environmental impact metrics for machine proxy datasets

1 $$\overline{{X }_{c}}= \frac{1 }{n} \sum_{i=1}^{n}{X}_{m}\;for\;subset\;of\;category\;\{c\}$$

The machine proxy data results, listed in full in.

Table 2, are used by simply multiplying each of the impact category results (Global warming, Stratospheric ozone depletion, Ionizing radiation…) by the mass in kilograms of the object for which an estimated environmental impact is sought as in Eq. 2.

**Table 2:** Machine proxy data result per kg of machine category

Impact category	Short name	Units	(Cluster 1) technological complexity	(Cluster 3) high complexity	(Cluster 2) low complexity
Global warming	*GWP*	kg CO₂ eq	20.152	8.088	5.359
Stratospheric ozone depletion	*ODP*	kg CFC-11 eq	1.01E-05	3.99E-06	1.86E-06
Ionizing radiation	*IR*	kBq Co-60 eq	1.764	0.409	0.268
Ozone formation, Human health	*POFP-HH*	kg NOₓ eq	0.065	0.020	0.014
Fine particulate matter formation	*PMFP*	kg PM10 eq	0.054	0.025	0.012
Ozone formation, Terrestrial ecosystems	*POFP-TE*	kg NOₓ eq	0.067	0.021	0.015
Terrestrial acidification	*TAP*	kg SO₂ eq	0.118	0.059	0.024
Freshwater eutrophication	*FEP*	kg P eq	2.73E-02	9.95E-03	4.32E-03
Marine eutrophication	*MEP*	kg N eq	1.07E-03	4.69E-04	3.29E-04
Terrestrial ecotoxicity	*TETP*	kg 1,4-DCB eq	369.344	259.796	63.058
Freshwater ecotoxicity	*FETP*	kg 1,4-DCB eq	15.060	5.326	1.525
Marine ecotoxicity	*METP*	kg 1,4-DCB eq	19.549	6.726	1.936
Human carcinogenic toxicity	*HTP-C*	kg 1,4-DCB eq	4.192	2.697	2.333
Human non-carcinogenic toxicity	*HTP-NC*	kg 1,4-DCB eq	194.581	53.951	16.604
Land use	*LU*	m^2^a	0.867	0.251	0.159
Mineral resource scarcity	*MRS*	kg Fe eq	0.834	0.283	0.146
Fossil resource scarcity	*FRS*	kg oil eq	5.263	1.909	1.348
Water consumption	*WC*	m^3^	0.209	0.084	0.048

Equation 2 Mass-based scaling of category-level machine proxy environmental impacts

2 $$Environmental\;Impact=Category\;Function \times Object\;Mass$$

### Evaluation of the practical utility of cluster-derived proxy datasets

Validation of proxy LCA datasets is inherently constrained by the limited availability of published machine-level LCAs that report results consistently across all 18 ReCiPe impact categories. In practice, most studies report only a subset of indicators—typically global warming potential—or present normalised results, limiting their suitability for direct comparison.

Accordingly, validation in this study is conducted internally by comparing the cluster-derived proxy datasets with the original machine-specific datasets after reassignment to their respective categories. The purpose is not to predict impacts for entirely new machine types, but to assess whether the proxy datasets provide a representative approximation of environmental profiles within each cluster. Deviations between individual machines and their corresponding category-level proxies therefore quantify the degree of generalisation introduced through clustering.

Datasets such as the *helicopter* highlight this issue, as they may initially appear misclassified; however, closer examination shows that the dataset represents a highly basic machine model composed of two metals with no further processing.

This approach does not constitute independent external validation and cannot fully evaluate transferability beyond the sampled population. In addition, because the proxies are derived from the same underlying data, some structural similarity is inevitable. Nevertheless, given the study’s objective of developing screening-level proxy datasets under typical LCA uncertainty ranges, this validation is sufficient to demonstrate practical applicability while clearly acknowledging its limitations.

The accuracy of the proxies ultimately depends not only on statistical aggregation but also on the classification of machines within categories and the interpretation of dataset descriptions. Certain datasets, for example those labelled as complex systems, may in practice represent simplified material compositions. The resulting proxy profiles are therefore influenced by definitional and interpretative choices. While minor variations in methodological parameters or classification decisions may affect the final results, substantial changes in overall conclusions are unlikely. A detailed sensitivity analysis of such interpretative variability is beyond the scope of the present study.

## Results and discussion

### Clustering of LCA data for manufacturing machines

**Fig. 2:**
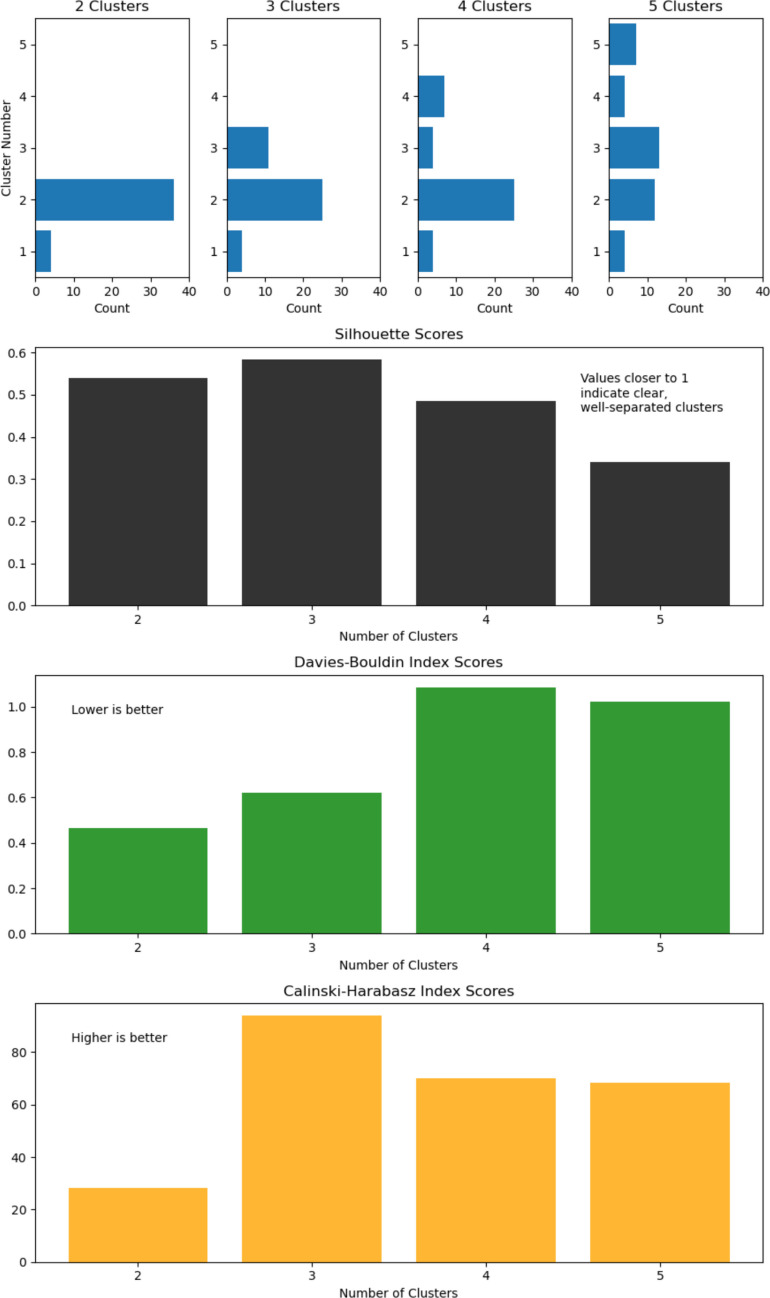
Internal validation metrics for hierarchical clustering, supporting a three-cluster solution

Cluster solutions were generated for between two and five clusters to assess how the structure of the dataset evolves as the level of partitioning increases. For each configuration, the spatial distribution of machines in the reduced feature space (PCA representation) was examined alongside standard clustering quality metrics. Figure 2 illustrates how machines are allocated under each clustering scenario, allowing visual assessment of separation, overlap, and internal cohesion.

The three-cluster solution provides a clear and interpretable grouping pattern, balancing separation and internal consistency without creating unstable or weakly populated clusters. The quantitative clustering metrics support this visual interpretation. A Silhouette Score of 0.584 indicates moderate separation between clusters. The Davies–Bouldin Index of 0.621 suggests relatively compact clusters with acceptable inter-cluster separation, as lower values indicate better-defined groupings. The Calinski–Harabasz Index of 93.92 further supports this configuration, reflecting dense clusters with substantial between-cluster dispersion relative to within-cluster variance.

Taken together, both the visual inspection and the clustering quality indices indicate that the three-cluster solution offers the most balanced and statistically coherent representation of the dataset among the configurations tested.

**Fig. 3:**
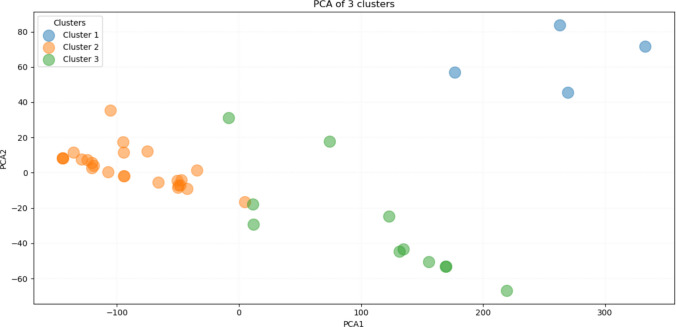
Principal component analysis of machine life-cycle impact datasets, illustrating similarity structure identified using Ward’s hierarchical clustering

Principal Component Analysis (PCA) is a dimensionality reduction technique that transforms a high-dimensional dataset into a smaller set of orthogonal components that capture the majority of the variance in the data. Figure 3 illustrates the distribution of datapoints along the first two principal components (PCA1 and PCA2) derived from hierarchical clustering using Ward’s linkage method.

Each point represents an individual machine positioned according to its scores on the principal components, where proximity between points indicates similarity in their underlying environmental impact profiles. The figure visually indicates the presence of well-defined clusters within this reduced-dimensional space.

### Definition of clusters into machine categories

Through analysis of the machines grouped within the three identified clusters, together with an assessment of the underlying data quality, three distinct machine categories have been derived based on relative homogeneity and engineering complexity.

While the present approach treats machines as homogeneous systems, more detailed representations could be developed by combining multiple proxy datasets to represent major component elements. Building on this framework, three broad categories of machine homogeneity are defined, capturing a progression from simple, uniform material compositions to increasingly complex machines characterised by specialised components, heterogeneous materials, and diverse manufacturing processes.

**Cluster 2** objects have an overall homogeneity that tends towards simple. They tend to be objects which have heavy bulk materials as part of their bill of materials, such that, though the object itself may have complexity, the bulk material takes up a greater proportion of the object when it is considered as single homogenous item.

**Cluster 3** objects are often mechanical devices which are more complex and more heavily reliant on electrical components. They are not necessarily in of themselves an electrical device, such as a mobile phone or laptop, but may be a device which produces work through the use of electrical components such as the vacuum cleaner.

**Cluster 1** objects appear to coalesce around items which are highly likened to digital technology, where the primary function of the object is not mechanical but electrical, such as a television.

Future research could refine this approach by identifying concrete, measurable physical or functional attributes that enable machine classification to be performed objectively and mathematically rather than qualitatively.

The resulting machine proxy data datasets are shown in.

Table 2 Overall, the results demonstrate a strong level of internal consistency and support the general robustness of the proxy dataset methodology. These results represent consolidated manufacturing datasets that capture the environmental performance of diverse machinery in a comparable form. Each dataset is based on embodied emissions, which is the impacts of producing the machine rather than the impacts related to the machines usage.

### Comparing results with ecoinvent data

The newly derived category-level proxy datasets are compared with the original machine-specific ecoinvent datasets to evaluate their suitability and to quantify the deviation introduced by generalising from detailed ecoinvent values. This comparison provides an indication of how closely the proxy datasets reproduce the environmental profiles of individual machines.

This validation approach is adopted because there is very limited published data reporting full environmental impact profiles for machines. In most cases, literature focuses on global warming potential or presents results normalised across the 18 ReCiPe impact categories to identify material or component hotspots, rather than reporting complete impact results.

The objective of this validation study is to confirm that the proxy datasets provide reasonable and realistic environmental estimates for machines that would be classified under the newly defined categories. In this context, some of the original datasets are less representative. For example, the helicopter dataset is grouped within (Cluster 2) Low Complexity due to the simplicity of the available ecoinvent data. However, in practical terms, helicopters are complex systems and would more appropriately be represented by a combination of (Cluster 1) Technological Complexity and (Cluster 3) High Complexity proxy data. In such cases, the proxy datasets are able to provide a closer approximation of real-world impacts than the existing ecoinvent representation.

**Fig. 4:**
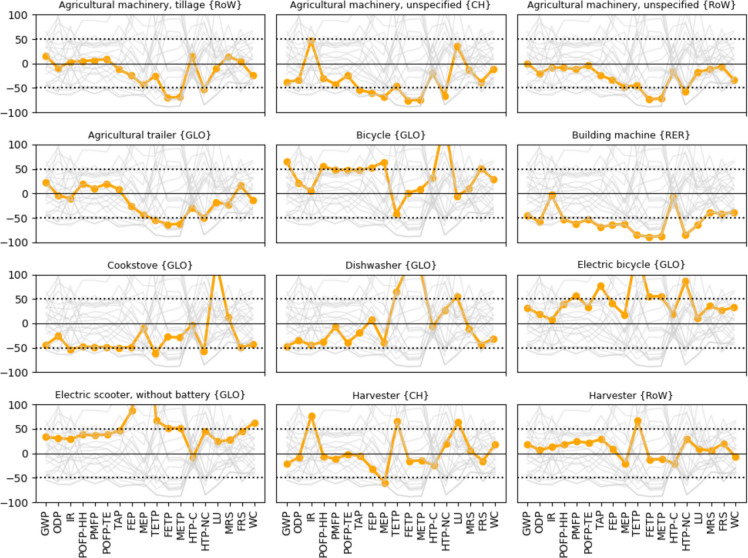
Comparison of first 12 machine-specific ecoinvent LCA results with category-level proxy datasets, expressed as percentage deviation across all ReCiPe environmental impact categories. Grey lines denote other machines within the same cluster for low complexity (Cluster 2) results

Low Complexity (Cluster 2) proxy data shows the greatest variability across the results as seen in Fig. 4. This is likely due to the larger number of machines included in this category. Overall, the Low Complexity proxy provides reasonable agreement with the corresponding ecoinvent profiles.

The results highlight the inherent variability associated with characterising the environmental profile of a machine across 18 impact categories. In the majority of cases, the machine-specific results fall within ± 50% of the corresponding proxy estimates, indicating a broadly acceptable level of agreement for screening-level applications.

However, isolated deviations are observed in certain instances. For example, the *Dishwasher {GLO}* dataset exhibits a single impact category in which the deviation exceeds 100%, extending beyond the plotted axis limits. Such outliers reflect the difficulty of capturing highly category-specific impacts within an aggregated proxy profile and underscore the trade-off between generalisation and precision when developing category-level datasets.

**Fig. 5:**
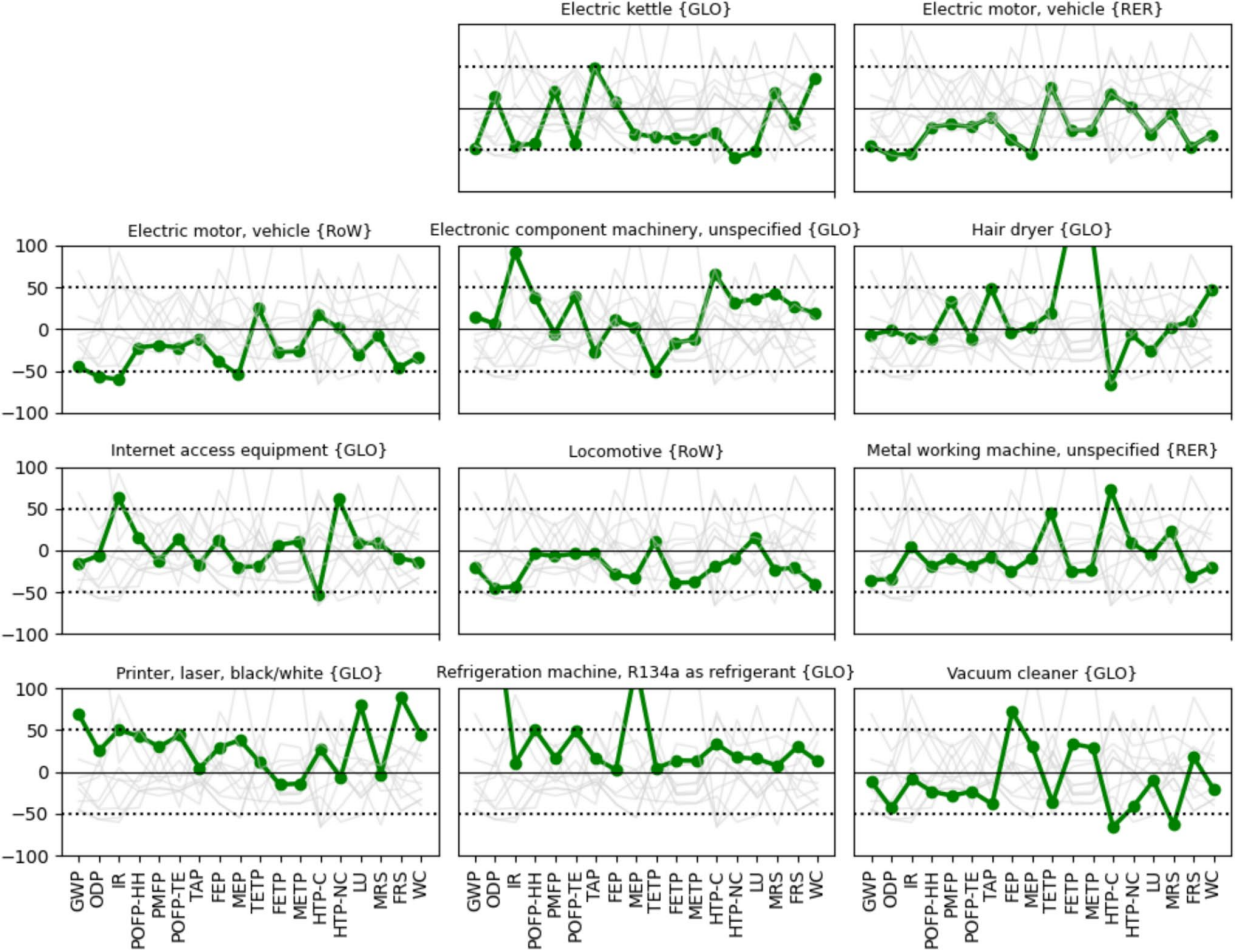
Comparison of machine-specific ecoinvent LCA results with category-level proxy datasets, expressed as percentage deviation across all ReCiPe environmental impact categories. Grey lines denote other machines within the same cluster for (Cluster 3) high complexity results

A similar pattern is observed for the (Cluster 3) High Complexity machine results as shown in Fig. 5. No individual machine aligns perfectly with the profile of its corresponding proxy dataset. Nevertheless, the proxies demonstrate generally strong performance as estimation tools at the screening level. A measurable range of deviation is observed across the 18 environmental indicators, which is expected given the breadth, differing units, and heterogeneous nature of the impact categories included within the ReCiPe framework.

Illustrative examples such as the *Electric kettle {GLO}* and *Internet access equipment {GLO}* show variation across individual impact categories, yet their results remain largely within ± 50% of the proxy values overall. This pattern reflects normal dispersion around a category mean rather than systematic misclassification.

There are, however, isolated cases of pronounced deviation in specific indicators. For example, the *Hair dryer {GLO}* exhibits a deviation exceeding 100% in the Marine ecotoxicity category. Such instances highlight the limitations of aggregated proxy profiles in capturing highly indicator-specific behaviour, particularly for impact categories that may be driven by small material fractions or specialised components.

**Fig. 6:**
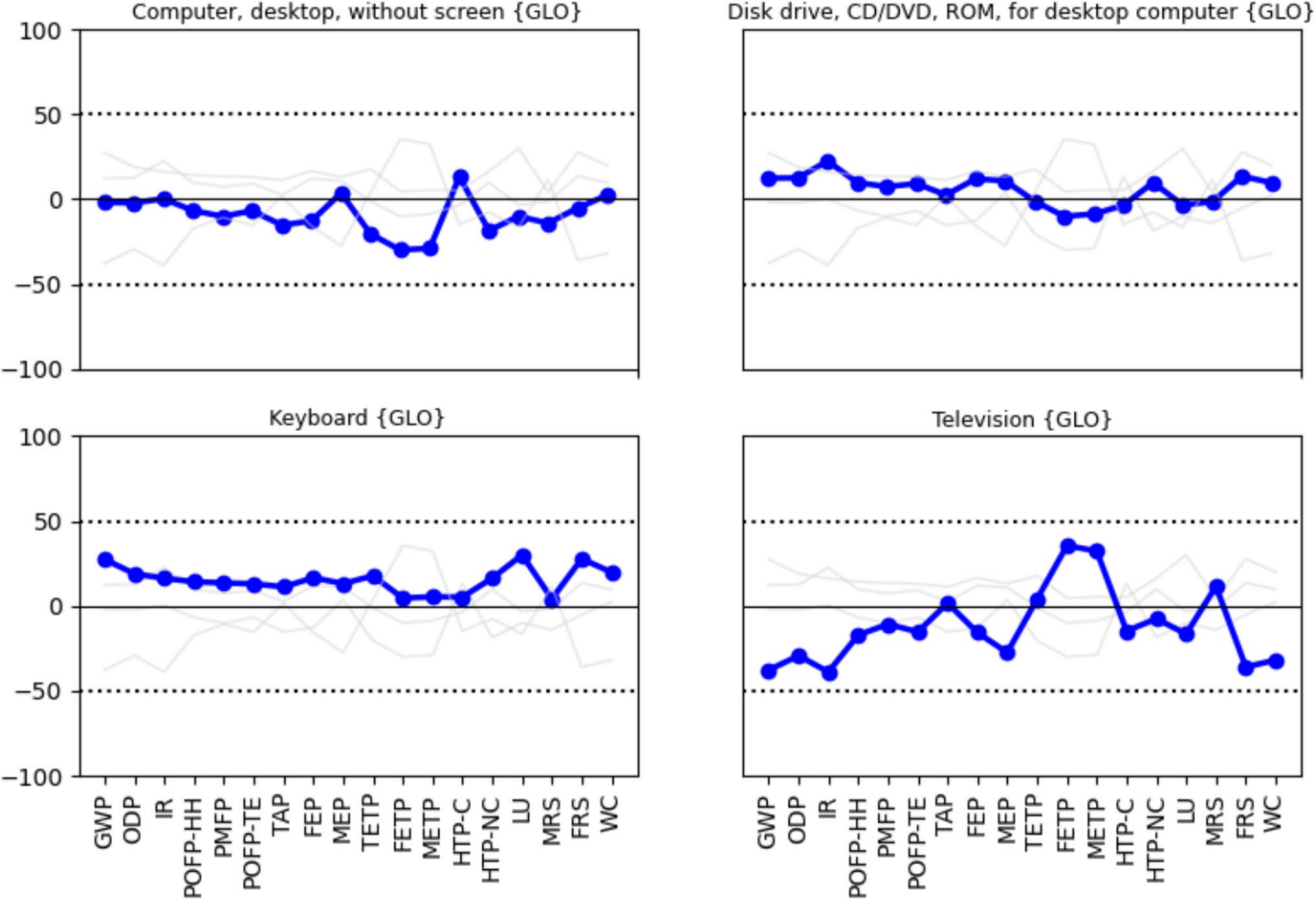
Comparison of machine-specific ecoinvent LCA results with category-level proxy datasets, expressed as percentage deviation across all ReCiPe environmental impact categories. Grey lines denote other machines within the same cluster for (Cluster 1) technology complexity results

The results for Cluster 1 (Technological Complexity machines) are comparatively more consistent than those of the other categories as shown in Fig. 6. This reflects the smaller number of machines within this cluster, which reduces internal heterogeneity and leads to a more coherent environmental profile. All machines assigned to this category fall within the ± 50% deviation bounds across the assessed impact categories, indicating that the proxy dataset provides a relatively stable and representative approximation for this group.

Overall, the comparisons indicate that the category-level values maintain reasonable agreement with the original machine-specific datasets. In most cases, the deviations observed are not substantial when considered in the context of the intended use of these datasets as generalised proxies, particularly given that uncertainties of up to approximately 40% are commonly reported even for more detailed LCA data. The resulting aggregated environmental profiles therefore provide a sufficiently representative depiction of machine impacts for screening-level and exploratory analyses. Consequently, the category values support rapid, high-level assessment of machine sustainability characteristics at a level of accuracy consistent with their intended application.

The results clearly illustrate the limitations of this approach and reinforce that these generalised values are not a substitute for detailed, machine-specific LCA studies. The observed deviations reflect the inherent variability of machine designs and material compositions, even within simplified categories. Increasing the number of categories could reduce deviation; however, doing so would undermine the practicality and simplicity of the method by increasing the assessment effort required for unknown machines.

## Further verification against non-studied machines

A verification study was also conducted using datasets independent of those used in the original proxy data development. Three low complexity, one high complexity and one technologically complex machines were evaluated against the new proxy datasets.

The life cycle inventory for *“Trawler, steel {RoW} | trawler construction, steel”* lists the primary materials and components associated with the construction of a steel trawler. However, the dataset appears to largely represent material inputs, while omitting explicit energy inputs related to manufacturing, processing, and assembly operations. Notably, the inventory does not include explicit energy inputs (e.g., electricity, fuels, or heat) which would be associated with machining processes or final assembly. As a result, the dataset primarily reflects the embodied impacts of material production rather than the full set of manufacturing activities required to construct the vessel.

**Fig. 7:**
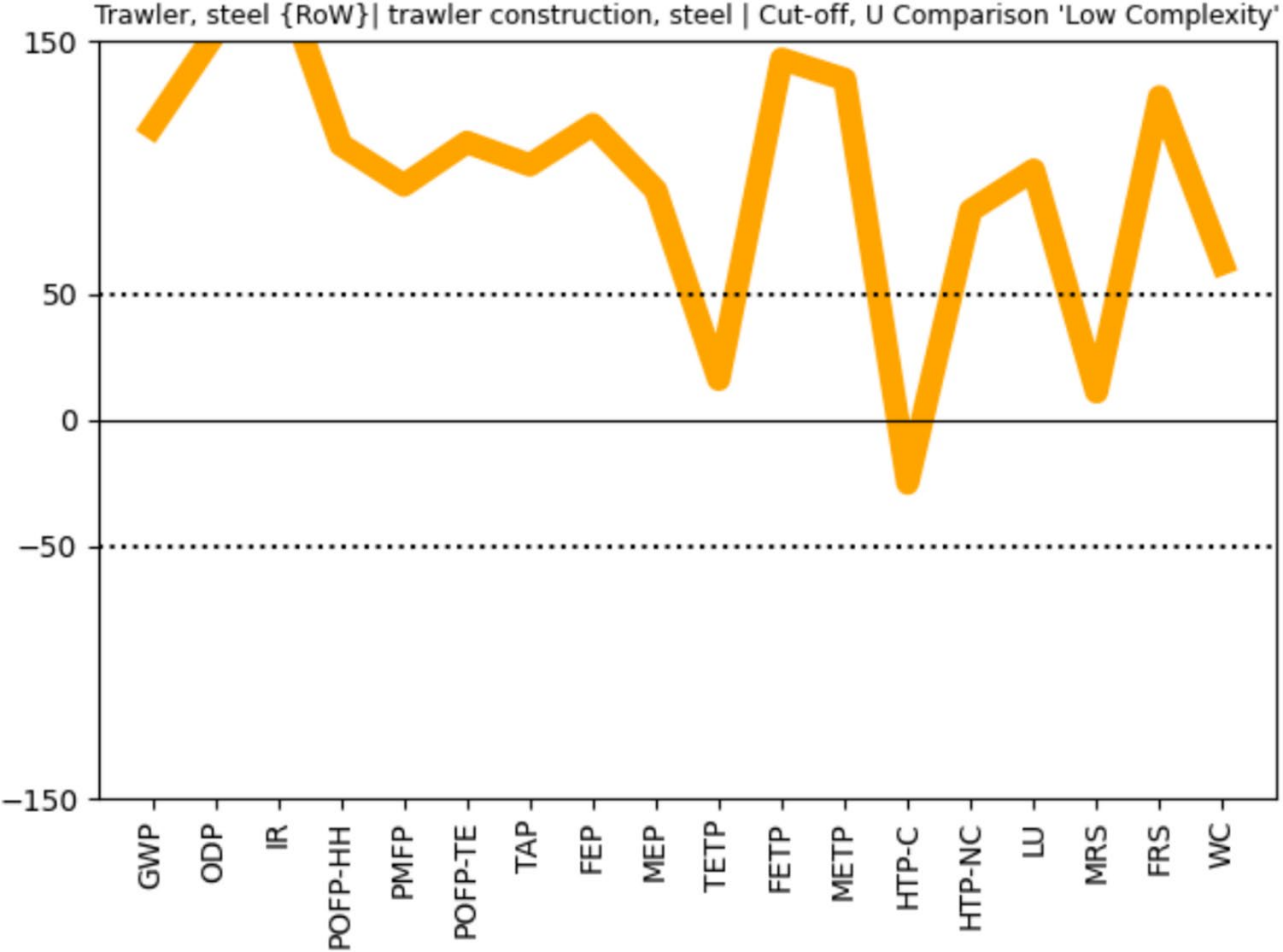
Comparison of trawler ecoinvent LCA results with category-level proxy datasets, expressed as percentage deviation across all ReCiPe environmental impact categories

Consequently, when applying the low-complexity proxy estimation approach, the resulting impact estimates are higher than those reported for the original ecoinvent dataset as shown in Fig. 7. If additional energy inputs representing fabrication and assembly processes were incorporated into the trawler dataset, the resulting life cycle impacts would be expected to increase and therefore converge more closely with the proxy-based estimates.

**Fig. 8:**
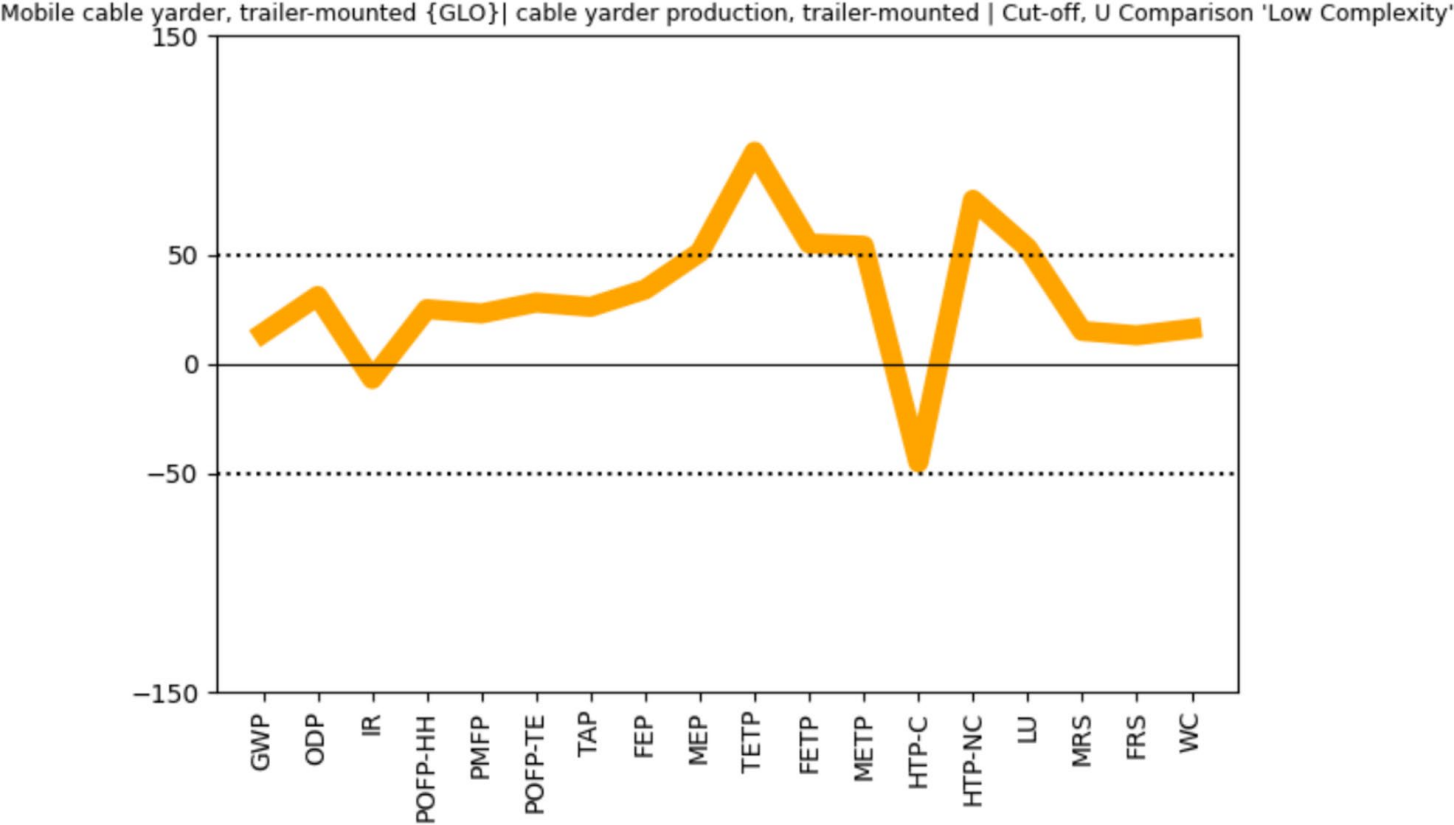
Comparison of mobile cable yarder ecoinvent LCA results with category-level proxy datasets, expressed as percentage deviation across all ReCiPe environmental impact categories

The “Mobile cable yarder, trailer-mounted {GLO}” dataset does include electricity and heat energies in the LCI for production and maintenance. The results shown in Fig. 8 show a closer relationship between the ecoinvent dataset and the new proxy dataset in this case.

**Fig. 9:**
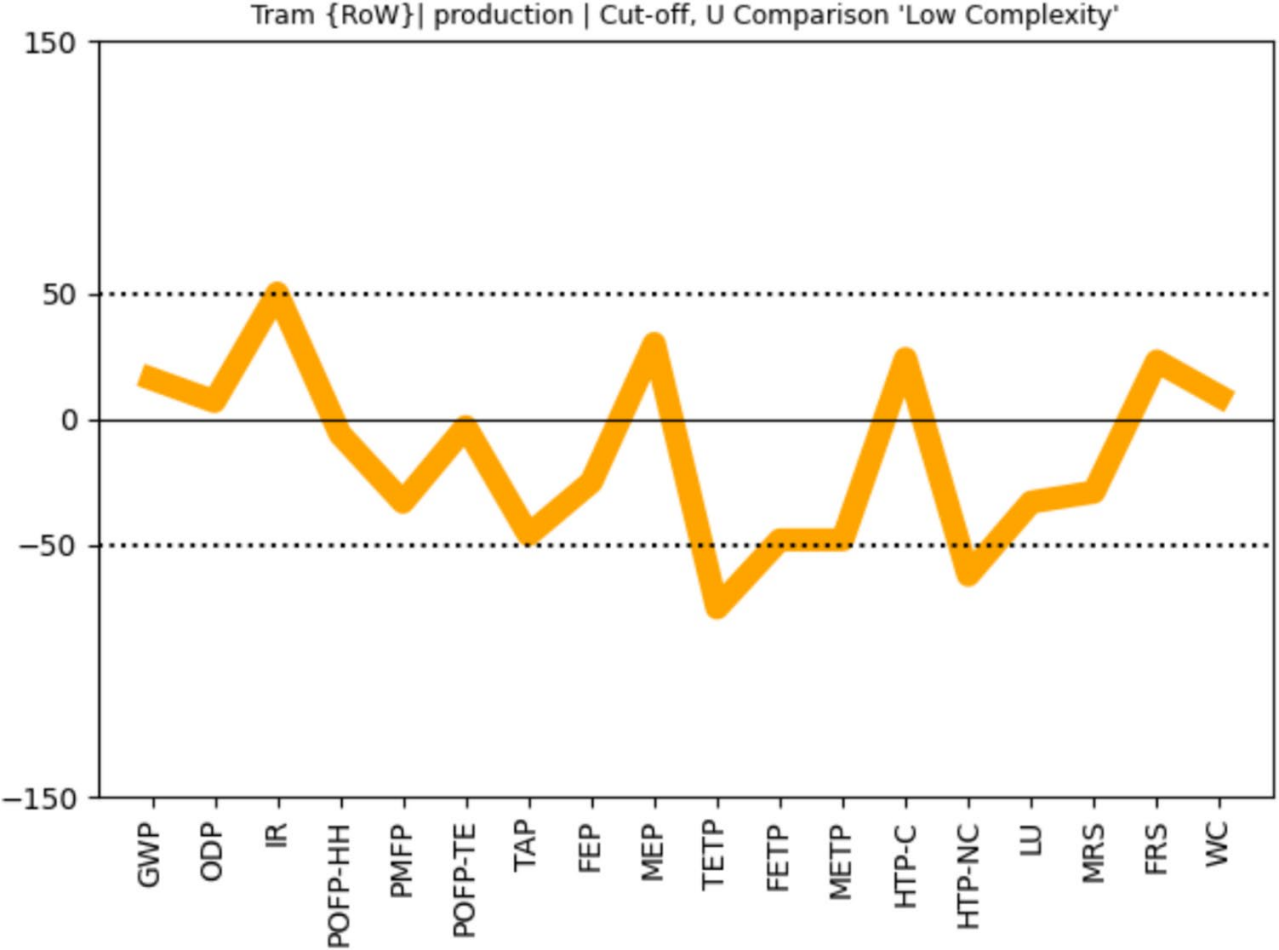
Comparison of Tram ecoinvent LCA results with category-level proxy datasets, expressed as percentage deviation across all ReCiPe environmental impact categories

Furthermore the data for the Tram {RoW} dataset which includes both materials and energy inputs agrees nicely with the low complexity proxy data derived in this study as shown in Fig. 9.

**Fig. 10:**
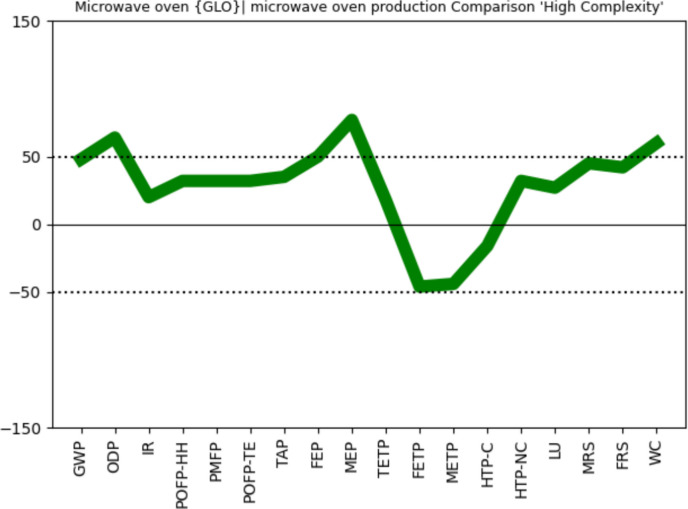
Comparison of microwave ecoinvent LCA results with category-level proxy datasets, expressed as percentage deviation across all ReCiPe environmental impact categories

Further examples of high-complexity machinery are comparatively difficult to identify within the ecoinvent database. Nevertheless, a comparison was conducted using a dataset representing a 10.6 kg microwave appliance. When evaluated against the high-complexity proxy dataset, the proxy approach provides a reasonably accurate estimation of environmental impacts as shown in Fig. 10.

Across the majority of impact categories, the calculated results fall within ± 50% of the values reported in the original dataset. This level of agreement suggests that, despite inherent simplifications, the high-complexity proxy method is capable of capturing the dominant contributors to life cycle impacts for such products with acceptable accuracy.

**Fig. 11:**
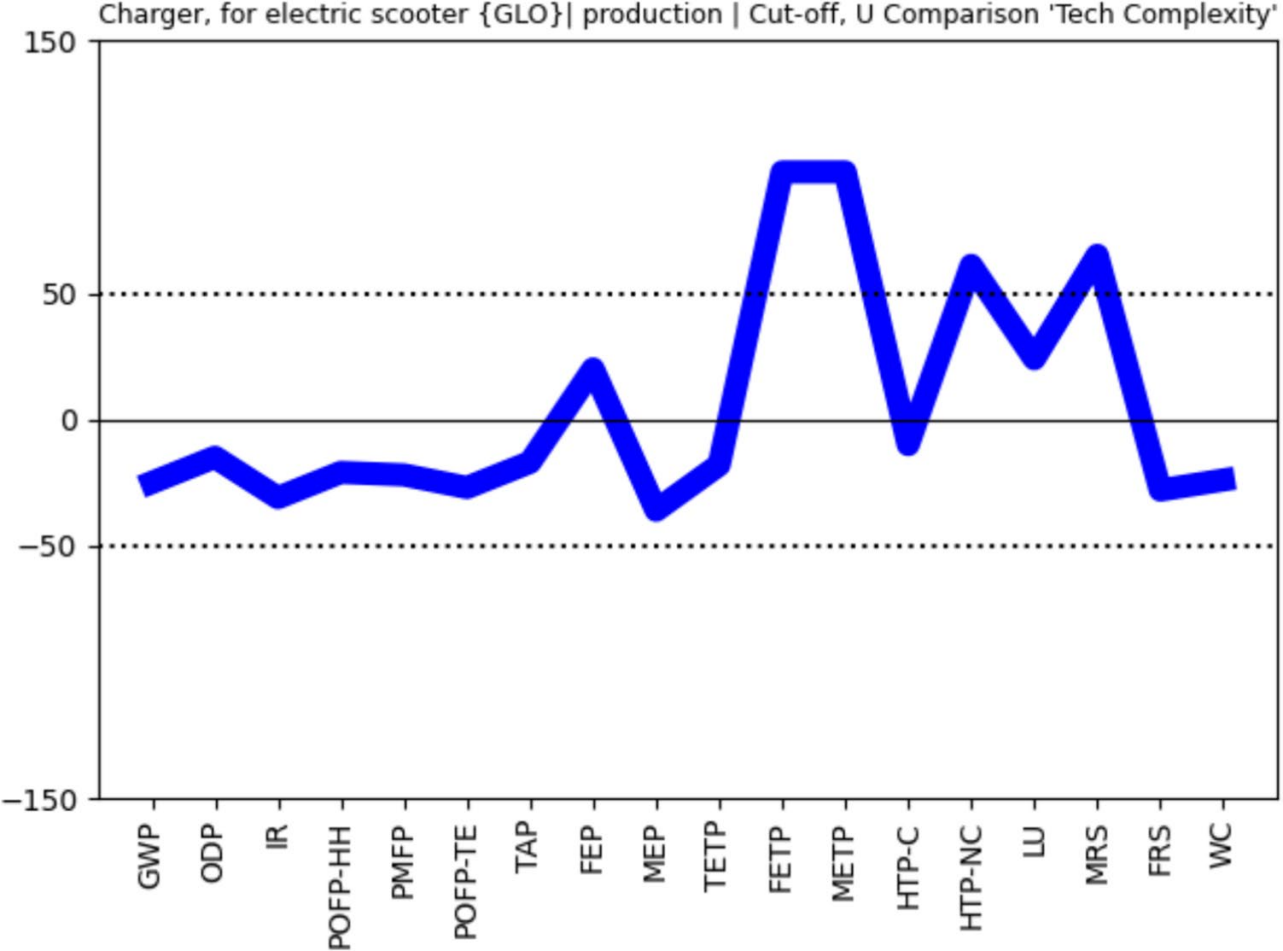
Comparison of charger ecoinvent LCA results with category-level proxy datasets, expressed as percentage deviation across all ReCiPe environmental impact categories

Finally, a comparison was performed between the technology-based proxy and a dataset representing an electric scooter charger (Fig. 11). The results indicate that the proxy approach provides a reasonably accurate estimation of environmental impacts for this product.

Technology-oriented proxies are generally more susceptible to finding devices outside of the scope of the proxies, as highlighted by the outliers identified in the original study, which were predominantly associated with highly technology-intensive products. These devices typically have significant contributions from printed electronic components.

In the case of the electric scooter charger, the relatively low proportion of printed electronics reduces this source of variability. Consequently, the technology-based proxy is better able to approximate the underlying life cycle impacts, resulting in a closer agreement with the reference dataset.

This additional verification study demonstrates that the proxy datasets provide useful and reasonably accurate estimations for machine and tool-like devices. However, given that the analysis spans 18 different environmental impact categories, and that underlying datasets vary in how they are sourced, modelled, and characterised, it is not feasible for a single proxy to perfectly represent all impact categories in every case. Despite these limitations, the proxy approach performs well overall. The results are not specific to the machines used to generate the original proxy data and are shown to be transferable to alternative equipment datasets, supporting their broader applicability.

## Conclusions

Performing a Life Cycle Assessment (LCA) is often a time-intensive and complex process, further complicated by challenges in locating suitable datasets. This issue is particularly relevant in engineering and manufacturing, where a diverse range of machines and tools require evaluation. Traditionally, practitioners face a trade-off: either conduct individual LCAs for each machine, which is resource-intensive, or rely on pre-existing datasets, which may not exist or be difficult to find and often contain limitations or inaccuracies.

This study demonstrates that it is feasible to cluster LCA datasets into meaningful industrial classifications. In this case, the objective was to identify groups of machines within the context of manufacturing, using a broad definition based on any mechanical object capable of producing work. From this analysis, three generalised LCA categories were produced. These are low complexity, high complexity and technologically complex machines, tools and devices. These categories are currently defined based on implicit similarities, but further research could identify specific, measurable features that differentiate machine types, enabling end users to generate LCA profiles for individual machines based on those characteristics.

The machine proxy data developed in this study addresses key challenges in conducting LCAs by enabling rapid and simplified estimations of a machine’s life cycle impacts, from production to end-of-life. This approach streamlines LCA decision-making, making it accessible even when detailed data is unavailable or incomplete. The proxy data serves as an effective screening tool, helping practitioners determine where detailed assessments are most needed, while providing manufacturers and engineers with a practical, ready-to-use resource for integrating environmental considerations into everyday decision-making. By reducing data barriers and enabling rapid assessments, this approach has the potential to accelerate sustainable manufacturing practices at scale in areas such as product design, equipment procurement, and process optimisation.

Validation of the proxy datasets against individual ecoinvent library results demonstrated that the category-level results provide reliable, high-level estimates within typical uncertainty ranges for generalised datasets. While these results are useful for screening and exploratory purposes, they further emphasise that generalised datasets cannot replace full LCAs when the goal is to accurately understand the environmental impact of a specific machine.

From an industrial perspective, the dataset could be integrated into sustainability assessment software used by manufacturers, procurement teams, and policymakers. Embedding the machine proxy data in digital twin models or automated LCA platforms would allow industries to perform quick sustainability assessments during machine selection, procurement, and operational planning. This would enable better decision-making, ensuring that environmental impact assessments are both efficient and scalable across diverse industrial applications.

## Figures and Tables

**Fig. 12:**
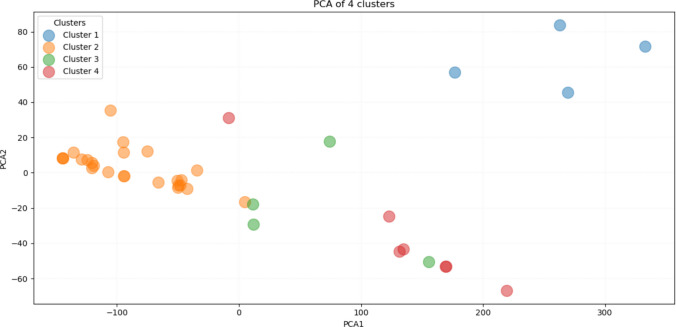
PCA based on 4 clusters

**Fig. 13:**
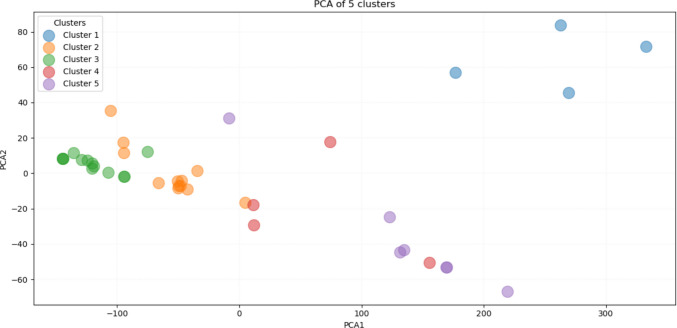
PCA based on 5 clusters

## Data Availability

The data used in this study are available from the ecoinvent database, version 3.11 (ecoinvent Association, Zurich, Switzerland). Access to the database requires a license, which can be obtained via https://ecoinvent.org.
